# Tissue and Temperature-Specific RNA-Seq Analysis Reveals Genomic Versatility and Adaptive Potential in Wild Sea Turtle Hatchlings (*Caretta caretta*)

**DOI:** 10.3390/ani11113013

**Published:** 2021-10-20

**Authors:** Julie C. Chow, Nia Kyritsis, Micah Mills, Matthew H. Godfrey, Craig A. Harms, Paul E. Anderson, Andrew M. Shedlock

**Affiliations:** 1 Genome Center, University of California, Davis, CA 95616, USA; julie.c.chow@gmail.com; 2 Program in Bioinformatics, Franklin and Marshall College, Lancaster, PA 17604, USA; nkyritsi@fandm.edu; 3 Program in Biological Sciences, Green Mountain College, Poultney, VT 05764, USA; micahmills90@gmail.com; 4 North Carolina Wildlife Resources Commission, Sea Turtle Project, Beaufort, NC 28516, USA; matt.godfrey@ncwildlife.org; 5 Duke Marine Laboratory, Nicholas School of the Environment, Duke University, Beaufort, NC 28516, USA; 6 Center for Marine Sciences and Technology, Department of Clinical Sciences, College of Veterinary Medicine, North Carolina State University, Morehead City, NC 28557, USA; caharms@ncsu.edu; 7 Department of Computer Science and Software Engineering, California Polytechnic State University, San Luis Obispo, CA 93407, USA; pauleanderson@gmail.com; 8 Department of Biology, College of Charleston, Charleston, SC 29424, USA; 9 College of Graduate Studies, Medical University of South Carolina, Charleston, SC 29425, USA; 10 Hollings Marine Laboratory, Marine Genomics Group, Charleston, SC 29412, USA

**Keywords:** differential gene expression, temperature-specific gene expression, *Caretta caretta*

## Abstract

**Simple Summary:**

Digital transcriptomics is rapidly emerging as a powerful new technology for modelling the environmental dynamics of the adaptive landscape in diverse lineages. This is particularly valuable in taxa such as turtles and tortoises (order Testudines) which contain a large fraction of endangered species at risk due to anthropogenic impacts on the environment, including pollution, overharvest, habitat degradation, and climate change. Sea turtles (family Cheloniidae) in particular invite a genomics-enabled approach to investigating their remarkable portfolio of adaptive evolution. Our de novo transcriptome assemblies and measurements of tissue- and temperature- specific global gene expression in the loggerhead sea turtle (*Caretta caretta*) reveal the genomic basis for potential resiliency in this endangered flagship species, and are crucial to future management and conservation strategies with attention to changing climates. We summarize the interactions among differentially expressed genes by producing network visualizations, and highlight the shared biological pathways related to development, migration, immunity, and longevity reported in the avian and reptilian literature. Our original results for loggerhead sea turtles provide a large, new comparative genomic resource for the investigation of genotype–phenotype relationships in amniotes.

**Abstract:**

**Background**: Digital transcriptomics is rapidly emerging as a powerful new technology for modelling the environmental dynamics of the adaptive landscape in diverse lineages. This is particularly valuable in taxa such as turtles and tortoises (order Testudines) which contain a large fraction of endangered species at risk due to anthropogenic impacts on the environment, including pollution, overharvest, habitat degradation, and climate change. Sea turtles (family Cheloniidae) in particular invite a genomics-enabled approach to investigating their remarkable portfolio of adaptive evolution. The sex of the endangered loggerhead sea turtle (*Caretta caretta*) is subject to temperature-dependent sex determination (TSD), a mechanism by which exposure to temperatures during embryonic development irreversibly determines sex. Higher temperatures produce mainly female turtles and lower temperatures produce mainly male turtles. Incubation temperature can have long term effects on the immunity, migratory ability, and ultimately longevity of hatchlings. We perform RNA-seq differential expression analysis to investigate tissue- and temperature-specific gene expression within brain (*n* = 7) and gonadal (*n* = 4) tissue of male and female loggerhead hatchlings. **Results**: We assemble tissue- and temperature-specific transcriptomes and identify differentially expressed genes relevant to sexual development and life history traits of broad adaptive interest to turtles and other amniotic species. We summarize interactions among differentially expressed genes by producing network visualizations, and highlight shared biological pathways related to migration, immunity, and longevity reported in the avian and reptile literature. **Conclusions**: The measurement of tissue- and temperature-specific global gene expression of an endangered, flagship species such as the loggerhead sea turtle (*Caretta caretta*) reveals the genomic basis for potential resiliency and is crucial to future management and conservation strategies with attention to changing climates. Brain and gonadal tissue collected from experimentally reared loggerhead male and female hatchlings comprise an exceedingly rare dataset that permits the identification of genes enriched in functions related to sexual development, immunity, longevity, and migratory behavior and will serve as a large, new genomic resource for the investigation of genotype–phenotype relationships in amniotes.

## 1. Introduction

Turtles and tortoises (Testudines) include some of the most endangered groups of vertebrates worldwide, yet have exhibited a remarkable resiliency in their long-term survival and adaptability to environmental change. New technologies for quantifying the differential gene expression in total cellular RNA are allowing us to more precisely model the dynamics of the turtle genotype–phenotype relationship and develop more predictive, genomics-enabled approaches to studies of turtle adaptive evolution and species conservation. Sea turtles (Cheloniidae) in particular have evolved a marine life history that includes an astonishing array of adaptive traits of broad interest to organismal biologists. As hatchlings, loggerhead sea turtles (*Caretta caretta*) emerge from their nests and enter the ocean, where they will forage as juveniles until maturing into bottom-feeding sub-adults and adults [1]. Adult loggerheads successfully navigate long distances between foraging habitats, wintering sites, and natal beaches to mate, with notable fidelity to migratory routes [2,3,4]. The large geographical range of the loggerhead sea turtle contributes to the species’ vulnerability to fisheries bycatch and habitat degradation. Loggerheads are classified as a vulnerable species with a decreasing population trend by the IUCN Red List (2017).

Loggerheads exhibit temperature-dependent sex determination (TSD), in which incubation temperature influences the primary sex ratio of developing offspring. For loggerheads, temperatures below the pivotal temperature of approximately 29 °C in the middle third of incubation produce mainly male hatchlings, whereas temperatures above 29 °C produce mainly female hatchlings [5,6,7]. With warming climates, there is a risk of skewed sex ratios in loggerhead populations which may negatively impact the persistence of global populations [8]. Elevated incubation temperature has been shown to negatively affect immune function, locomotion, and ultimately survival in turtles [9,10,11,12]. Phenotypic plasticity in nesting phenology may combat short-term effects of environmental warming, but continued adaptation of loggerhead populations to shifting thermal conditions may not be sustainable in the long-term.

Previous studies in turtles and other reptiles have identified differentially expressed genes at female and male promoting temperatures [13,14,15,16], and enrichment of sex-specific and heat shock proteins were detected recently in loggerheads [17,18]. We present a summary of the application of an RNA-seq analysis pipeline to a rare dataset consisting of brain and gonadal tissue from experimentally reared female and male loggerhead hatchlings. Through enrichment analyses, we identify biological processes and significantly differentially expressed genes potentially relevant to sexual development, migration, longevity, and immunity within our study against the reptile and avian literature. An improved understanding of differential gene expression at a critical developmental period in loggerhead sea turtles may reveal genes that may contain genetic variation important to adaptive potential in changing environments thereby extending the predictive value of genomic resources for managing loggerheads [19]. 

## 2. Methods

### 2.1. Hatchling Sample Collection and RNA Extraction

A visual summary of our sampling design and analytical workflow to identify significantly differentially expressed genes related to TSD, migration, and longevity via genome-wide transcriptomic analysis of loggerhead brain and gonadal tissue from male and female turtles is displayed in Supplemental file S6.

A total of 11 *C. caretta* hatchlings were used in our transcriptomics study, which were originally used as part of a pivotal temperature experiment from the Northern Recovery Unit of the Southeastern United States [20]. In the summer of 2011, 7 eggs from which brain tissue would be harvested were salvaged from the same nest, and similarly, 4 eggs from which gonadal tissue would be harvested were collected from the same nest in the summer of 2013. We were unable to collect brain and gonadal tissue from the same animals because other samples had been assigned to different collaborative studies [21,22,23] to maximize the information derived from these rare samples. The eggs were collected from “doomed” nests that would not have been successful if left in place due to erosion and or predation. We followed the accepted protocols of the American Veterinary Medical Association (AVMA) Guidelines on Euthanasia (June 2007 edition, current at the same of sample collection and consistent with the 2020 edition), and used decapitation followed immediately by pithing to euthanize hatchlings. Full permit information is listed in the Acknowledgements.

Salvaged eggs were transported <12 h after laying for laboratory incubation at the NCSU Center for Marine Sciences and Technology (CMAST) and reared in temperature-controlled incubators above and below 29 °C for expected female and male development, respectively [20]. From the total sample of 11 individuals, brain tissue was collected from 4 male and 3 female hatchlings, and gonad tissue was collected from 2 male and 2 female hatchlings. Sex was confirmed histologically at CMAST by previously published methods [20,21,22,23,24].

Brain and gonad tissue were immediately placed into RNA*later* reagent (Qiagen, Inc., Germantown, MD, USA) for high quality RNA stabilization after dissection. Tissues were homogenized and purified, and cDNA was extracted using Qiagen TissueRuptor, RNeasy, and Omniscript Reverse Transcription (RT) kits. RNA samples were quantified via an Agilent Bioanalyzer prior to Illumina TruSeq library preparation. Brain samples were single-end sequenced on an Illumina HiSeq 2000 (1 × 100 bases) and gonad samples were paired-end sequenced on an Illumina HiSeq 2500 (2 × 100 bases) at the Medical University of South Carolina (MUSC) Center for Genomic Medicine.

### 2.2. De Novo Transcriptome Assembly

The quality of RNA sequenced ends was assessed using FastQC (http://www.bioin-formatics.babraham.ac.uk/projects/fastqc/, accessed on 19 October 2021), and low-quality reads were discarded. Reads were then trimmed via Trimmomatic (0.32) (-phred33) with LEADING:3, TRAILING:3, SLIDINGWINDOW:4:15, and MINLEN:36 for brain and MINLEN:70 for gonads [25]. De novo transcriptome assembly for brain and gonad reads was completed using the Trinity software package (r20140717) using separate references for brain and gonads with default parameters and a minimum contig length of 400 [26], resulting in one brain and one gonad assembly.

### 2.3. Differential Gene Expression and Enrichment Analyses

Differential gene expression in brain and gonad tissues among male and female log-gerheads were evaluated using the RSEM-EBSeq pipeline [27,28]. Transcript reads were provided to RSEM (1.2.18) and aligned to the transcriptome assembly via Bowtie2 (--bowtie2) using default parameters [27,29].

Clustered heatmaps were constructed via the analyze_diff_expr.pl script (-P 0.001 -C 2 –max_DE_genes_per_comparison 50) from Trinity. Transcripts were annotated via NCBI BLASTX (2.2.31) using non-redundant proteins from vertebrates with -max_target_seqs 3 and -evalue 0.0001, and significantly differentially expressed transcripts (q-value < 0.05, in which q-value refers to FDR corrected *p*-values) were retained.

Gene ontology (GO) enrichment was performed via OmicsBox (1.2.4) [30]. To perform enrichment analysis with OmicsBox, Blastx-fast was executed through CloudBlast with default parameters using non-redundant protein sequences (nr_v5). To further functionally characterize the protein families, domains, and sites associated with the assembled sequences, InterProScan was run within OmicsBox, enabling by default the use of ProfileScan, HMMSmart, HMMTigr, Gene3D, SLFD, SuperFamily, and MobiDBLite databases. Following annotation, Fisher’s Exact Test (FDR < 0.05) was implemented to identify significantly enriched terms with default OmicsBox settings.

Significant GO terms (FDR < 0.05 and e-value < 1.0 × 10^−6^) were retrieved and visualized via REViGO with default settings (allowed similarity = medium; 0.70) [31]. To functionally cluster transcript annotations, DAVID (6.8) was used on significantly differentially expressed transcripts with default settings [32]. Protein–protein interaction networks of significantly differentially expressed genes were constructed via Cytoscape (3.8.0) and the stringApp plugin (1.5.1) with a confidence score 0.7 to select for high confidence interactions and *Chelonia mydas* as the species of interest [33]. The Network Analyzer tool within Cytoscape was used to calculate node degree per gene and identify hub genes and other network statistics. Genes with a degree greater than 3 were visualized within constructed interaction networks. Functional enrichment was assessed via the STRING enrichment function.

## 3. Results

To characterize differential gene expression relevant to the development of broad life history traits, including immunity, migratory behavior, and longevity in loggerhead sea turtles (*Caretta* *caretta*) subjected to temperatures below and above the pivotal temperature (29 °C) for sex determination, we performed RNA sequencing and de novo assembly of the transcriptomes of female and male loggerhead hatchlings using brain (4 male, 3 female) and gonadal (2 male, 2 female) tissue. Significantly enriched gene ontology terms were identified, and potential association of differentially expressed transcripts with temperature-dependent sex determination (TSD), migration, longevity, and immune function were identified by comparison with genes previously reported in the reptile and avian literature, including those for reference genome assemblies published for turtles [34,35].

**Figure 1:**
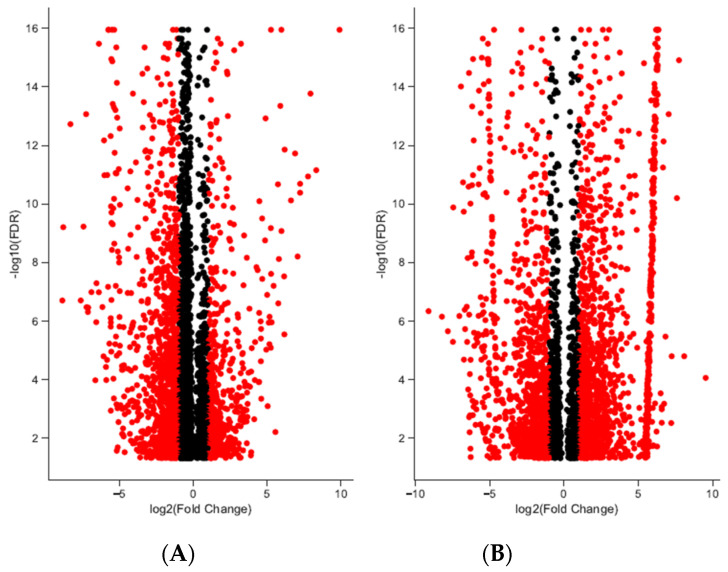
Differentially expressed transcripts in brain and gonadal tissue in loggerhead sea turtles reared below and above 29 °C. Significant differentiation from fold change (FC) of two is indicated in red (FDR < 0.001). In brain (**A**), 774 and 1513 transcripts are significantly up- and downregulated with fold change greater than 2; 1270 and 872 transcripts with fold change greater than 2 are significantly up- and downregulated in gonads (**B**).

**Figure 2:**
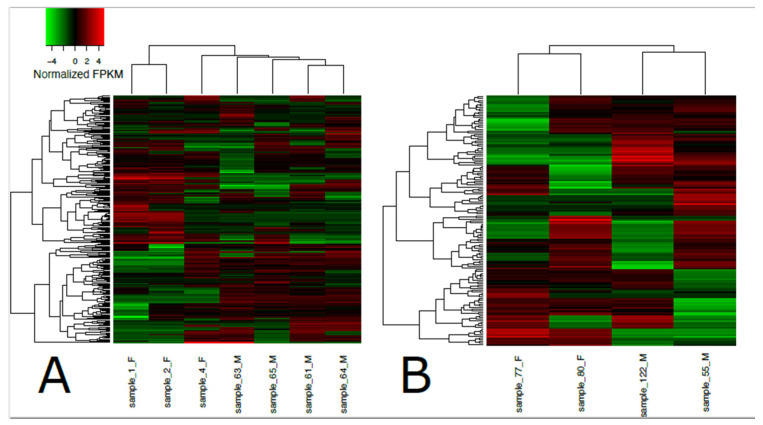
Heatmap of differentially expressed transcripts in loggerhead brain (**A**) and gonadal (**B**) tissue clustered by sex. Sex is indicated in sample names for females (F) and males (M). Significantly expressed transcripts (FDR < 0.001) with at least 4-fold differential expression are displayed in normalized FPKM values using the top 50 differential expression features within pairwise comparisons.

A total of 192,076 transcripts were identified for loggerhead brain tissue, and 150,489 transcripts were identified for gonadal tissue (Table 1). Approximately 96.31% and 97.14% of transcript reads passed trimming quality filters with an average length of 1399 and 1733 bp, respectively, for brain and gonad. In brain tissue from male and female loggerheads, 1513 and 774 transcripts were found to be significantly downregulated (PostFC < 0.5) and upregulated (PostFC > 2), respectively, at false discovery rate (FDR) < 0.001; in gonad, 872 transcripts were found to be downregulated, while 1270 were found to be upregulated (FDR < 0.001, PostFC < 0.5 and > 2) (Figure 1). Selected candidate genes potentially relevant to TSD, immunity, migratory behavior, and other broad life history traits that are significantly differentially expressed in female and male loggerhead brain and gonadal tissue are displayed in Table 2. Hierarchically clustered heatmaps of tran-scripts with at least 4-fold differential expression (FDR < 0.001) while extracting the top 50 differential expression features per pairwise comparison (--max_DE_genes_per_compar-ison 50) are displayed in Figure 2 for loggerhead brain and gonad.

### Gene Ontology Enrichment and Functional Annotation Clustering

To identify pathways and biological processes related to TSD, migration, longevity, and immunity, Gene Ontology (GO) enrichment was performed via OmicsBox 1.2.4. A total of 3629 transcripts were assigned GO terms for brain, and 3289 GO terms were assigned for gonad, of which 2149 and 994 biological process GO terms displayed significant (FDR < 0.05) enrichment in brain and gonadal tissue, respectively. Selected biological processes relevant to sexual development are displayed in Table 3. Biological processes related to cellular metabolism and DNA metabolism (GO:0006259) were significantly en-riched in brain, as were the terms DNA repair (GO:0006281) and the cell cycle (GO:0007049) in gonadal tissue (Supplemental file S1). Overall, the enrichment of terms relevant to transport (GO:0006810) and biosynthetic processes in brain and the enrichment of chromosomal organization (GO:0051276), and signal transduction (GO:0007165) in gonadal tissue suggests the enrichment of processes important for embryonic development. REViGO was used to summarize enriched GO terms by reducing functional redundancies, producing treemaps indicating that protein phosphorylation and metabolism-related biological processes are enriched in the loggerhead brain and gonads (Supplemental files S2 and S3).

Functional annotation was performed via DAVID to identify shared enrichment of biological functions among significantly differentially expressed genes (q-value < 0.01). Functional analysis identified 1 enriched KEGG pathway (cmy04512:ECM-receptor interaction) in gonadal tissue and 2 KEGG pathways (cmy03460:Fanconi anemia pathway 13, cmy03440:Homologous recombination) in brain tissue with Benjamini-Hochberg < 0.05 (Supplemental file S4). In brain, shared functions of DNA repair and replication exists within the Fanconi anemia pathway 18 and Homologous recombination clusters, including shared enrichment of genes relevant to DNA polymerase and topoisomerase. The ECM receptor interaction cluster identified in gonads consists primarily of genes associated with collagen, laminin, and integrin, indicating an enrichment in signal transduction pathways and cellular communication in these tissues. The DAVID functional annotation clustering tool was used to further describe similarity among enriched functional annotations terms, identifying 11 clusters from brain and 8 clusters from gonadal tissue, and returning enrichment scores describing the degree of gene co-association; the clusters with highest enrichment scores for brain were associated with DNA repair and transport, and clusters relevant to metabolism were identified in gonads (Supplemental file S4).

**Figure 3:**
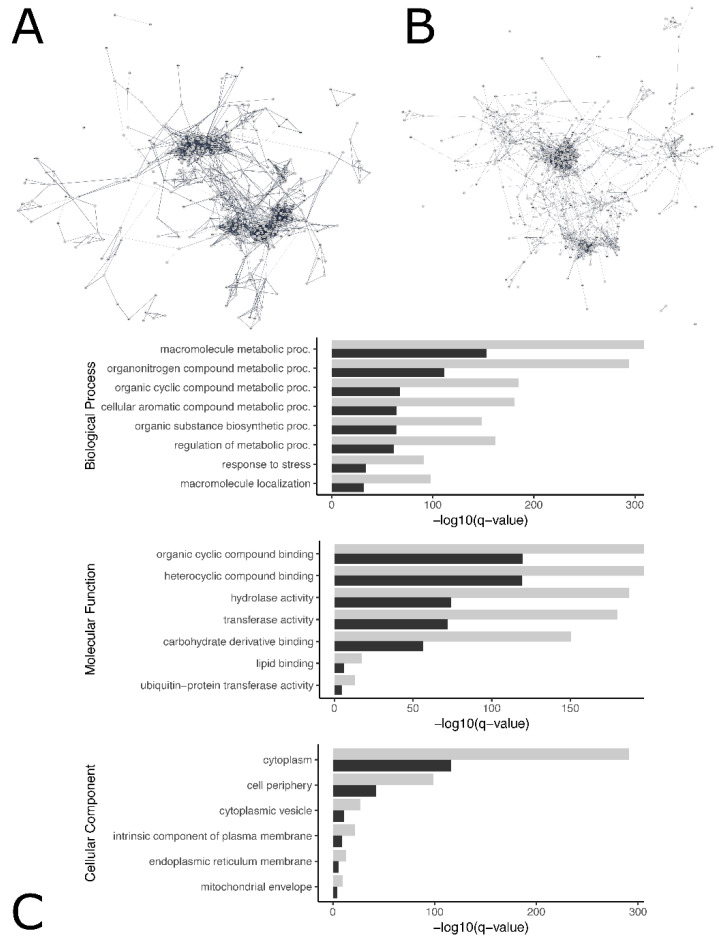
Visual representation of protein–protein interaction in brain (**A**) and gonads (**B**) for genes with degree greater than 3. Interactive visualizations of the constructed networks are publicly available for brain (https://www.ndexbio.org/viewer/networks/e3fedc13-18cf-11ec-9fe4-0ac135e8bacf, accessed on 19 October 2021) and gonads (https://www.ndexbio.org/viewer/networks/e3fedc13-18cf-11ec-9fe4-0ac135e8bacf, accessed on 19 October 2021). Gene-specific information, including association with enrichment terms can be viewed by selecting individual genes in the network. Larger degree indicates greater number of physical interactions between proteins associated with a gene and its neighbors in the network, and genes with large degree may be relatively important to multiple biological pathways. Topological parameters measuring node degree distribution, centrality, and other metrics were calculated and displayed per gene in Supplemental file S5 for gonads and brain. Significant biological processes, molecular function, and cellular process Gene Ontology (GO) terms associated with differentially expressed genes in brain (grey) and gonadal (black) tissue (**C**).

Protein–protein interaction networks of significantly differentially expressed genes among female and male loggerhead brain and gonadal tissue were constructed via Cytoscape to identify networks of interconnected genes potentially involved in the same biological pathways, identify genes of high degree likely to be important in multiple pathways relevant to temperature-specific sexual development, and visualize protein interactions (Figure 3). Constructed networks are publicly available on the Network Data Exchange (NDEx) Public Database, displaying gene-specific information including measures of centrality, degree, and associated functional enrichment terms (brain: https://www.ndexbio.org/viewer/networks/68a25356-18d2-11ec-9fe4-0ac135e8bacf, accessed on 19 October 2021; gonad: https://www.ndexbio.org/viewer/networks/e3fedc13-18cf-11ec-9fe4-0ac135e8bacf, accessed on 19 October 2021). A network with a total of 2502 nodes with 3309 edges was constructed to represent protein–protein interaction in brain tissue. The genes PDCD11, CDK1, and BRIX1 had the highest degrees observed. For gonads, a network of 2296 nodes and 3653 edges was created; CDK1 and CDC20 displayed the highest degrees. Node statistics for brain and gonad protein–protein interaction networks are displayed in Supplemental file S5. The stringApp was used to extract functional enrichment terms associated with groups of genes within the constructed protein–protein interaction networks, yielding significant enrichment in the coiled coil (KW-0175) in both gonads (q-value < 5.86 × 10^−7^) and brain (q-value < 2.9 × 10^−6^), and transferase (KW-0808, q-value < 1.5 × 10^−3^) and ECM-receptor interaction KEGG pathway (cmy04512, q-value < 3.47 × 10^−2^) in gonads (Supplemental file S5).

## 4. Discussion

### Biological Significance of Differentially Expressed Genes among Female and Male Loggerheads

We have performed de novo transcriptome assembly and differential gene expression analyses using brain and gonadal tissue from loggerhead hatchlings experimentally reared below and above the pivotal temperature for sex determination (29 °C). Significantly differentially expressed genes in gonadal tissue were generally enriched for signal transduction, while brain tissue was enriched in terms related to protein binding, transport, metabolism, and biosynthesis.

With special attention to genes potentially involved in the traits of TSD, migratory behavior, longevity, and immunity, we have identified significantly differentially ex-pressed genes of interest in loggerheads reared below and above the pivotal temperature for sex determination. Here, we highlight these genes from our analyses on loggerhead brain and gonadal tissue that have also been reported in the reptile and avian literature as pertinent.

*Temperature-dependent sex determination (TSD).*

Loggerheads display TSD, in which individuals reared above and below the pivotal temperature of 29 °C mainly develop into females and males, respectively. In both brain and gonads, the gene *HSD17B7* was found to be significantly differentially expressed. *HSD17B7* has previously been potentially associated with TSD and has a role in the synthesis of sex steroids [34,36,37].

*Genes differentially expressed in gonads relevant to TSD*. 

Candidate sex determining genes such as *FOXL2*, *RSPO1*, and *ESR1* were significantly enriched in gonadal tissue and tend to be highly expressed during female promoting temperatures in various turtle species [38,39,40,41]. However, the exact roles of certain genes that are important to gonadogenesis, such as *CBX2*, *EMX2*, *GATA4*, and *LHX9*, are relatively uncharacterized in TSD [14]. Our analysis further identified *SERPINH1, AMHR2*, and *BCR* as differentially expressed in loggerhead gonads, with potential functions in gonadogensis as suggested previously [42]. *AMHR2* is a TSD regulator, and *BCR* has been associated with stress and immune response. *CIRBP* was previously found to be involved in TSD in the snapping turtle (*Chelydra serpentina*) [42].

*Genes differentially expressed in brain relevant to TSD*. 

The genes KDM3A, important in ovary and testicle formation in turtles that exhibit TSD, an estrogen receptor interactor *GREB1* [43], a gene related to fetal sex in humans *GRB10*, a germ cell-specific factor necessary for meiotic progression *TOPAZ1* [44], and *M1AP*, a gene implicated in male germ cell development [45], were found to be significantly enriched in brain tissue. The role of *KDM6B* in TSD has been previously described in loggerheads, and *JARID2* has been found to be relevant to TSD via RNA-seq analysis of brain tissue in loggerheads and gonadal tissue in *Alligator mississippiensis* [16,17,46,47,48]. *STIP1* has been associated with heat shock proteins under thermal stress in loggerhead brain tissue, and the genes *A2ML1* and *ASB16* have also been found to be differentially expressed in the loggerhead brain during heat stress [17].

*Migration.*

From birth, loggerhead hatchlings migrate long distances from coastal areas to the open sea [49]. Loggerheads display natal homing behavior; many mature loggerheads forage in neritic zones close to natal nesting sites, and female loggerheads return to their natal nesting region to reproduce [1,50]. Previous evidence suggests that sea turtles are able to detect and distinguish among magnetic fields, enabling navigation [51]. Philopatry and the ability to successfully navigate long distances has been observed in many species [52]. The genes *MICAL1*, involved in FAD binding, and *IFT20*, both of which were significantly enriched in loggerhead brain, may play a role in photoreceptor activity and magnetic receptions in birds [53]. Additionally in the loggerhead brain or gonadal tissue, *CACNA1B*, *SRPK2*, *COL1A2*, *NRXN1*, *ARPP21*, and *ITPR1* were previously found to be significantly differentially expressed among juvenile and adult migrating willow warblers [54]. *NPAS2* and *DRD4* are associated with avian migratory behavior; specifically, *NPAS2* is associated with circadian rhythm, and *DRD4* is a candidate for exploratory behavior [55]. *VPS13A*, previously linked to avian migration, was enriched in loggerhead brain tissue [56].

*Longevity and immunity.*

The lifespan of a loggerhead can surpass 70 years (https://www.fisheries.noaa.gov/species/loggerhead-turtle, accessed on 19 October 2021), and the resiliency of the loggerhead immune system contributes to loggerhead longevity. Previously, *RHOF* has been implicated in normal B-cell function and is upregulated in malignant lymphoid tissue [57]. *SHISA6* regulates stem cell differentiation in spermatogonia by inhibition of Wnt [58]. *UBE3D* facilitates protein degradation and may be relevant to prevention of cellular senescence. Genes involved in tumor neovascularization and tumor suppression such as *SART3* and *NEDD4*, were found to be significantly differentially expressed in the loggerhead brain or gonads. There was significant differential expression of genes regulating apoptosis (*CCAR2*, *GRB10*), the cell cycle (*SIPA1*, *CCAR2*), and DNA repair (*RAD54B*), in addition to macrophage stimulation (*MST1R*, *CSF1R*) and other genes involved in immune cell signaling (*TLR3*, *IRAK2*, *BCAP29*).

## 5. Conclusions

We have characterized the differential expression of genes with potential relevance to TSD, migration, longevity, and immunity in brain and gonadal tissue of female and male loggerhead sea turtles. Significantly differentially expressed genes associated with gonadogenesis, synthesis of sex hormones, and gamete formation were identified as potentially relevant to TSD. A search of differentially expressed genes in male and female loggerheads identified genes in the avian and reptile literature with roles in magnetoreception, migratory behavior, cellular senescence, DNA repair, and immunity, among other functions.

We observed significant enrichment of biological processes specifically related to TSD and sexual development, indicating that distinct groups of genes in brain and gonads are differentially regulated in loggerheads experimentally reared below and above the pivotal temperature for sex determination in *C. caretta*. Through functional clustering and protein–protein interaction enrichment analyses, enrichment of the ECM-receptor pathway was identified in both brain and gonadal tissue, suggesting that extracellular communication may play an important role in sex determination.

Through summarization of differentially expressed genes in male and female loggerheads, this work has produced a set of candidate genes with particular relevance to mechanism of TSD. Further study of these genes may yield a clearer understanding of genotype–phenotype relationships and potential adaptive resiliency of endangered loggerheads worldwide and, by extension, other TSD species. We anticipate this new transcriptomic resource integrated with genome-wide surveys of locus-specific polymorphism [19] can inform future conservation efforts that will seek to address growing sex imbalances and survivorship in sea turtle populations experiencing warming climates.

## Figures and Tables

**Table 1:** Raw reads retrieved per sample and number of reads aligned to assembled transcriptomes for loggerhead brain and gonadal tissues.

Sample ID	Sex	Raw Reads	Reads Mapped to Transcriptome (%)
Brain			
1	Female	123,316,446	91.57%
2	Female	73,620,245	91.48%
4	Female	65,569,443	91.44%
61	Male	102,155,296	91.22%
63	Male	17,845,489	90.39%
64	Male	96,913,331	91.43%
65	Male	102,423,395	91.20%
Gonad			
77	Female	51,399,125	82.33%
80	Female	50,746,307	83.58%
55	Male	46,557,205	83.88%
122	Male	11,018,312	84.06%

**Table 2:** Genes with potential relevance to TSD, immunity, migratory behavior, and longevity in brain and gonadal tissue of loggerheads. Select genes with significant differential expression (FDR corrected *p*-value (q-value) < 0.05) and fold change greater than 2 among loggerheads reared below and above 29 °C are displayed.

**Brain**				
Accession	Gene	Description	q-value	Fold change
XP_007072694	SEPT1	septin 1	0	727.96
XP_008170752	LOC101945970	shugoshin-like 2	1.75 × 10^−2^	3.17
XP_007061912	MAPKAPK3	MAP kinase- activated kinase 3	9.67 × 10^−3^	5.58
XP_008177295	ESRRG	estrogen-related receptor gamma isoform X3	2.23 × 10^−2^	6.53
XP_007054941	JARID2	Jumonji isoform X1	4.49 × 10^−11^	2.09
XP_007060139	TOPAZ1	testis- and ovary- specific PAZ domain-containing 1	2.41 × 10^−5^	23.65
XP_007063347	GREB1	GREB1 isoform X1	4.01 × 10^−2^	14.79
XP_007063772	KDM3A	lysine-specific demethylase 3A	0	61.13
XP_007055513	RPS6KA5	ribosomal S6 kinase alpha-5 isoform X1	2.90 × 10^−3^	10.60
XP_008174364	KDM6B	lysine-specific demethylase 6B	5.60 × 10^−10^	3.13
XP_007056370	HSD17B7	3-keto-steroid reductase	3.77 × 10^−2^	5.31
XP_007069240	MSMP	prostate-associated microsemino	2.83 × 10^−4^	12.36
**Gonad**				
Accession	Gene	Description	q-value	Fold change
XP_007060743	RSPO1	R-spondin-1 isoform X2	4.63 × 10^−3^	5.51
XP_005283194	CBX2	Chromobox homolog 2	1.20 × 10^−2^	2.39
XP_005282573	FOXL2	forkhead box L2	0	64.96
XP_008170093	GATA4	transcription factor GATA-4 isoform X2	3.09 × 10^−2^	8.95
XP_007065874	AMHR2	anti-Muellerian hormone type-2 receptor	1.22 × 10^−6^	5.59
XP_006124717	LHX9	LIM homeobox Lhx9 isoform X4	1.40 × 10^−11^	3.88
XP_007063819	BCR	breakpoint cluster region isoform X1	7.92 × 10^−4^	2.18
XP_007065642	DMRT1	doublesex and mab-3 related transcription factor 1	1.67 × 10^−3^	70.35

**Table 3:** Significantly enriched biological processes in brain and gonadal tissue of loggerheads. Selected biological process Gene Ontology (GO) terms with significant enrichment between loggerheads reared below and above 29 °C are displayed. Q-value refers to *p*-values adjusted for the false discovery rate (FDR).

Biological Process Term	GO:ID	Brain q-Value	Gonad q-Value
Development of primary sexual characteristics	0045137	2.5527 × 10^−^^4^	2.2747 × 10^−^^2^
Embryo development ending in birth or egg hatching	0009792	4.8274 × 10^−^^7^	3.7780 × 10^−^^3^
Female gamete generation	0007292	2.2815 × 10^−^^2^	
Female sex differentiation	0046660	1.1049 × 10^−^^2^	
Germ cell development	0007281	5.4465 × 10^−^^5^	2.7750 × 10^−^^3^
Gonad development	0008406	2.5527 × 10^−^^4^	2.2747 × 10^−^^2^
Male gamete generation	0048232	1.1888 × 10^−^^4^	1.1216 × 10^−^^3^
Male sex differentiation	0046661	4.6000 × 10^−^^2^	
response to steroid hormone	0048545	4.4088 × 10^−^^8^	8.4302 × 10^−^^4^
Sex differentiation	0007548	1.1821 × 10^−^^4^	2.7750 × 10^−^^3^
Steroid biosynthetic process	0006694	1.1049 × 10^−^^2^	2.2747 × 10^−^^2^

## Data Availability

Original DNA sequence data for this project are openly available in the NCBI Biosample repository for DNA and RNA Resources under the SRA accession PRJNA663187.

## Supplementary Materials

The following are available online at https://www.mdpi.com/article/10.3390/ani11113013/s1, Supplemental file S1 (XLS). Enriched Gene Ontology terms in loggerhead brain and gonadal tissue. Enriched GO term categories are indicated and sorted by increasing p-value and FDR. Supplemental file S2 (PDF). REViGO Gene Ontology treemap. A summary of significantly enriched Gene Ontology terms from loggerhead brain tissue. Supplemental file S3 (PDF). REViGO Gene Ontology treemap. A summary of significantly enriched Gene Ontology terms from loggerhead gonadal tissue. Supplemental file S4 (XLS). Functional annotation and clustering of differentially expressed genes in loggerhead brain and gonadal tissue. Functional annotation terms are shown in ‘annotation’ tabs, and identified clusters are shown in ‘clustering’ tabs in order of decreasing enrichment score. Supplemental file S5 (XLS). Network parameter statistics per gene in loggerhead brain and gonadal tissue. Measures of node degree distributions, centrality, connectivity, and topological coefficients are displayed per gene in brain and gonad networks constructed from significantly differentially expressed genes. Supplemental file S6 (PDF). Visual summary of experimental design. RNA-seq sampling of hatchling transcriptomes per treatment and tissue type (brain, gonad) are summarized. A graphical sequence of sample-specific RNA processing for loggerhead hatchlings experimentally reared at the pivotal temperature for sex determination (29 °C), de novo transcriptome assembly, gene annotation, and differential gene expression analysis is displayed.

## Abbreviations

FDR	False discovery rate
GO	Gene Ontology
TSD	Temperature-dependent sex determination
