# Supplementary figures and images for: Tissue and Temperature-Specific RNA-Seq Analysis Reveals Genomic Versatility and Adaptive Potential in Wild Sea Turtle Hatchlings (Caretta caretta)
Source: Animals (Basel). 2021 Oct 20;11(11):3013. doi: 10.3390/ani11113013 (PMC8614379; doi:10.3390/ani11113013)

REVIGO Gene Ontology treemap

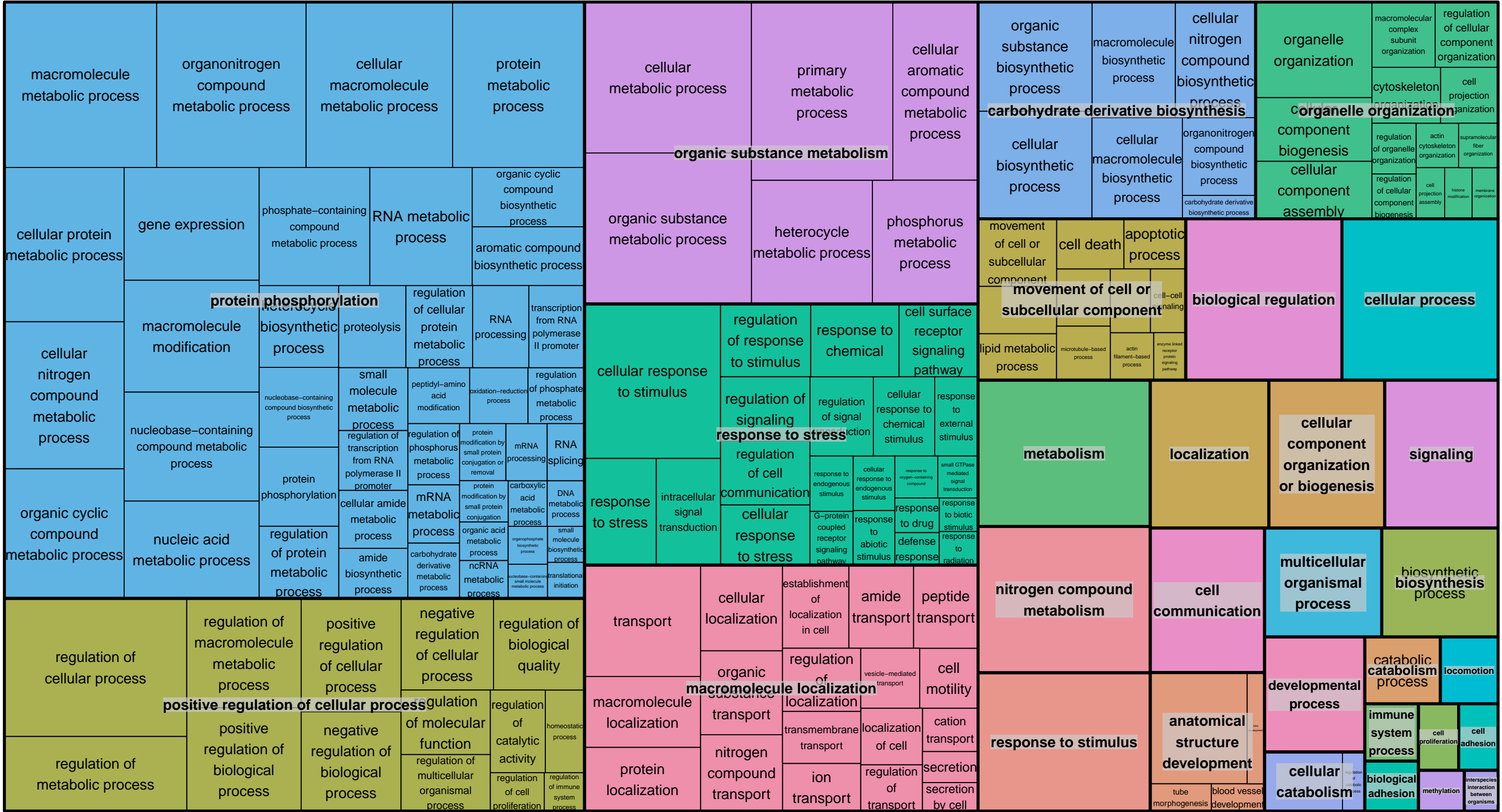

Supplement: Supplementary file 1 [file animals-11-03013-s001.zip › Supplemental File2.pdf]

## REVIGO Gene Ontology treemap

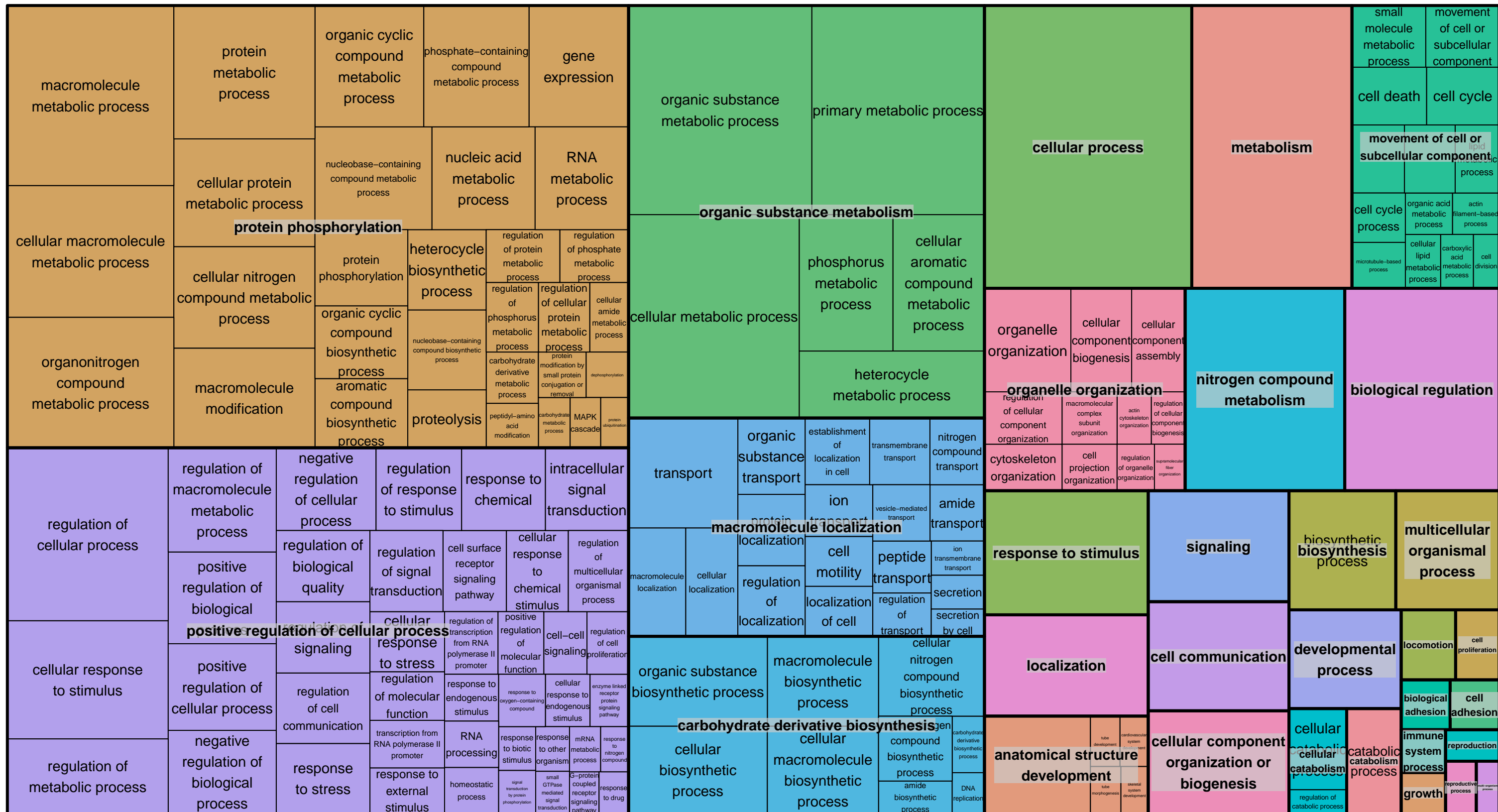

Supplement: Supplementary file 1 [file animals-11-03013-s001.zip › Supplemental File3.pdf]

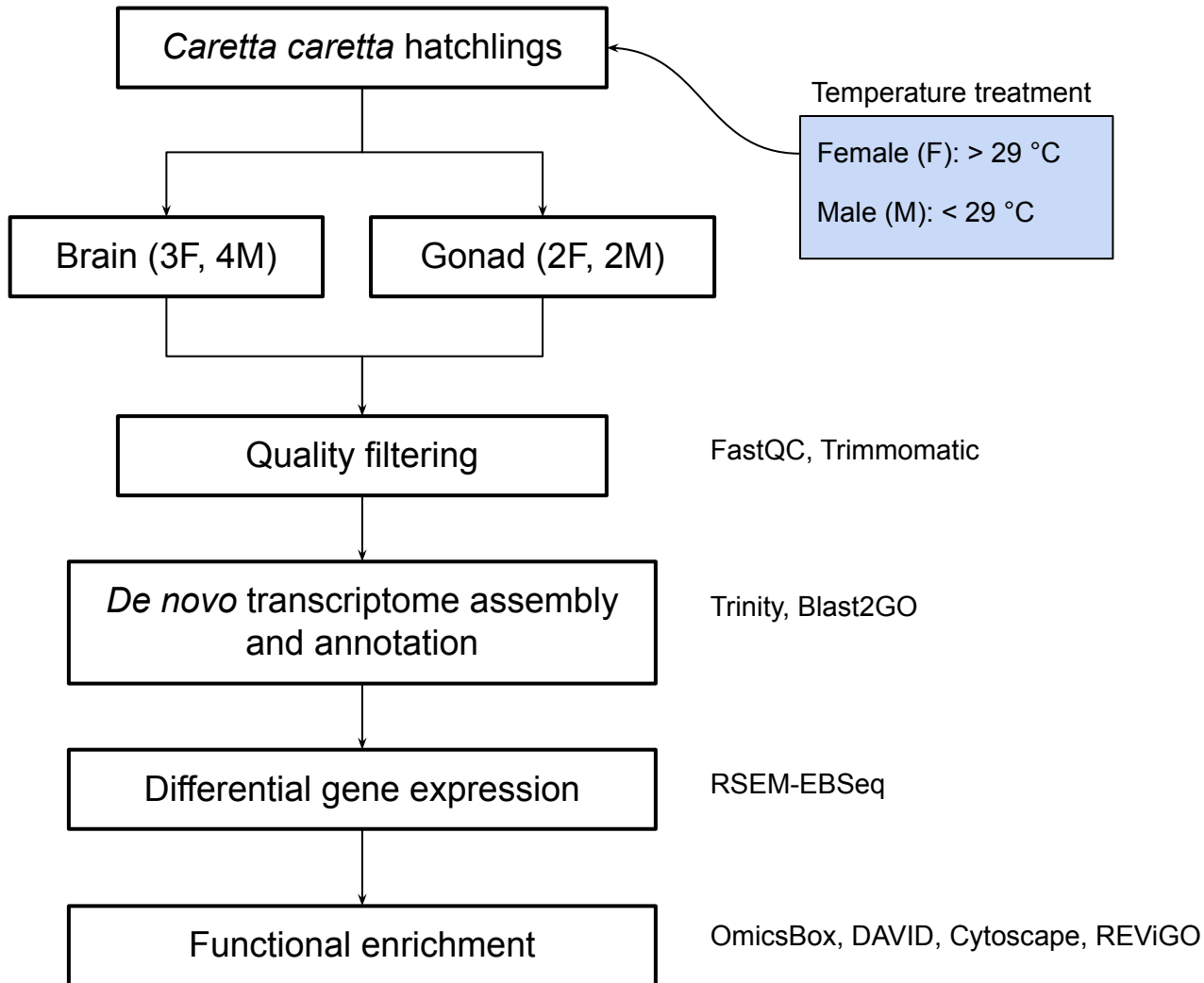

Supplement: Supplementary file 1 [file animals-11-03013-s001.zip › Supplemental File6.pdf]
